# Exploring learner engagement with languages (LX) within and beyond the English classroom

**DOI:** 10.1177/13621688231216869

**Published:** 2023-12-19

**Authors:** Giulia Sulis

**Affiliations:** University of Graz, Austria

**Keywords:** engagement with language, engagement with LX, language learning engagement, language repertoire, middle school, multilingualism

## Abstract

Given the complex language repertoire of a large number of students within Austrian schools, particularly in lower secondary schools, it would be critically important to understand their practices, attitudes, and beliefs towards the multiple languages they encounter in their different areas of life. In this study I redefine and expand on the construct of engagement with language to incorporate an investigation of the different languages (hereafter LX) that learners come into contact with in and outside of school. I propose the construct of ‘engagement with LX’ to depict how learners utilize, reflect on, and relate to the LX in their repertoires in all contexts of their lives, including English as a language of formal foreign language instruction. Participants in this study were nine learners from the same English class in an Austrian middle school. Data for this study were collected using a biodata questionnaire, classroom observations, video-audio recordings of the lesson, and semi-structured interviews. Findings revealed the complexity of learners’ multilingual lives within and beyond the classroom, as well as the interconnections between these domains. Findings have also shed light on the ways learners’ engagement with LX beyond the classroom can support their learning in the English classroom, and the kind of affordances for language learning they perceive across their multiple contexts. The study also offers practical implications in terms of how teachers can engage with learners’ whole LX repertoire to support their learning process.

## I Introduction

In recent years, Austria has faced several challenges in reducing the achievement gap between students with a migratory background (i.e. those who have migrated into a new country of residence and/or have at least one of their parents who did so; European Commission, 2023) and their non-migrant peers (OECD, 2017). In light of these concerns, the European Commission (2017) has recommended that Austria should ‘take steps to improve the educational achievements of disadvantaged young people, in particular those from a migrant background’ (p. 12). The NMS (*Neue Mittelschule* or ‘new middle school’) school type, more recently referred to as *Mittelschule* or MS (Bundesministerium für Bildung, Wissenschaft, und Forschung, 2020), was introduced in 2007/08 in Austria (OECD, 2017) as a way to provide comprehensive lower secondary education for students from diverse backgrounds. Unlike the academically oriented track (*Allgemeinbildende Höhere Schule*; AHS), whose attendance is contingent upon students’ grades in the last year of primary school, MS is open to all students after primary school (Herzog-Punzenberger & Schnell, 2019). While this type of lower secondary school was found to provide a more positive learning environment and better student support compared to general secondary schools, it has not shown any significant improvement in student outcomes (Eder et al., 2015). Indeed, recent statistics have revealed that, especially in urban settings, the achievement rates of students in MS schools were lower compared to the national average and much lower compared to their peers in general academic secondary schools (BIFIE, 2020). Furthermore, compared to other types of institutions, MS schools have a larger than average population of students from a migrant background (26%), and of students who do not typically speak German at home (24%); this percentage is much higher in large cities, reaching 60% in Vienna (BIFIE, 2020; Erling, Radinger, & Foltz, 2023; Statistik Austria, 2019). Crucially, the learning outcomes of learners who do not speak German at home were found to be lower compared to their peers who spoke German at home (BIFIE, 2020) across multiple school subjects including English, which is one of the core subjects in Austrian schools (OECD, 2016).

In light of the complex linguistic repertoire of a large number of students within Austrian schools, and particularly in MS schools (Statistik Austria, 2023), it would be critically important to understand how they relate to and use the multitude of languages they encounter within and beyond school, including English as the target of foreign language instruction. Here I refer to an individual’s linguistic repertoire as a set of skills and knowledge one has of one or more languages to any level of competence and mastery, and for different uses (Council of Europe, 2023). Earlier studies have shown that multilingual speakers employ their whole language repertoire to communicate in effective ways (e.g. Cenoz & Gorter, 2017). Research also suggests that, when English language teachers draw on their students’ existent linguistic knowledge and experiences from previous language learning, their learning outcomes can be enhanced (Otwinowska, 2017). As such, understanding learners’ engagement with all the languages learners come into contact with across different domains of their lives can contribute towards a better appreciation of how language skills and knowledge acquired outside the instructional context can be transferred to the classroom. Furthermore, it can also provide insights into how teachers can draw on students’ linguistic repertoires to support their language learning process in the English language classroom.

In this study, I redefine and expand on the construct of engagement with language (Svalberg, 2009) to investigate how learners in MS schools engage with languages in their lives more broadly, in order to gain a better understanding of how they interact with and use their different languages across their different contexts, how they perceive and make use of affordances to enhance their learning, and how language learning outside the classroom can support their learning in the English language classroom. The choice of the English classroom as one of the focal contexts of the study is twofold. As mentioned earlier, English is one of the core subjects in Austrian schools (OECD, 2016), and is the language chosen by over 91% of students in Austria to fulfil at least one of their foreign language requirements (Eurostat, 2019, as cited in Erling, Brummer, & Foltz, 2023). However, learners’ English outcomes in MS across Austria are below the national average (BIFIE, 2020). Furthermore, despite calls to integrate multilingual pedagogies and reconceptualize traditional models of language education, this is not a reality in most classrooms (Herzog-Punzenberger et al., 2017). Research by Erling, Radinger, and Foltz (2023) has highlighted that, English teachers in MS schools in Austria ‘do not seem to be engaging with students’ entire language repertoire to consolidate learning’ (p. 12). As such, there is a need to better understand how the English classroom can become ‘a space where students’ multilingualism and multilingual identities are validated’ (Erling, Brummer, & Foltz, 2023, p. 73).

### 1 Engagement with languages (LX)

Research on engagement has emerged from different theoretical traditions, contexts, and foci (Fredricks et al., 2016). Within applied linguistics specifically, the available body of empirical research has mainly addressed two areas. The first area builds upon earlier work in educational psychology and focuses on learners’ interaction with and reaction to learning activities, where language is not necessarily seen as the object but as a medium of communication (Philp & Duchesne, 2016). The second area concerns engagement with language, which was originally developed by Svalberg (2009) and defined as ‘a cognitive, and/or affective, and/or social process in which the learner is the agent and language is object (and sometimes vehicle)’ (p. 2). The focus of this study will be on engagement with language but with two key differences in respect to Svalberg’s (2009) original conceptualization of the construct.

First, instead of separating the agent (i.e. the learner) from the object (i.e. the language), the languages in a learners’ repertoire are seen in this study as an integral part of learners’ selves and identities. Furthermore, while engagement with language has predominantly focused on second language (L2) learning (e.g. Baralt et al., 2016), this study examines how learners utilize, reflect on, and relate to all the languages they come into contact in the different domains of their lives within and beyond the context of instruction. The term ‘LX’ was originally defined by Dewaele (2018) as ‘any foreign language acquired after the age at which the first language(s) was acquired, that is after the age of 3 years, to any level of proficiency’ (p. 3). Given the complex language repertoire of multilingual individuals, discerning between their first language (L1) and other LX can pose several challenges; as explained by Hammarberg (2010), ‘the problem is that it will often be neither meaningful nor even possible to order a multilingual’s languages along a linear time scale’ (p. 93). As such, to avoid this distinction, I employ the term ‘LX’ to refer to any language in a person’s language repertoire, regardless of the date of acquisition, level of mastery, and type of use, may it be their L1, L2, L3, etc. By taking a holistic perspective, the proposed study addresses learners’ whole linguistic repertoire to understand more broadly how they engage with the multitude of languages they encounter at home, at school, and beyond.

### 2 Three dimensions of engagement with LX

Engagement with LX is viewed in this study as a multidimensional construct. Engagement with language was originally conceptualized as including cognitive, affective, and social dimensions (Svalberg, 2009). However, in this study, all levels of engagement are understood as socially embedded; thus, the social component is seen as integrated in all dimensions of engagement. Furthermore, I also acknowledge a behavioural dimension of engagement with LX; as explained by Mercer et al. (2021), ‘to communicate, to learn the language by using it, the individual must be actively engaged in that process’ (p. 146).

In the proposed construct of engagement with LX, behavioural engagement is viewed in terms of actively using one’s whole LX repertoire. Much research has stressed the importance of ‘learning-by-doing’ when it comes to language development (e.g. Ellis et al., 2013), foregrounding the role played by oral and written production. In this study, however, behavioural engagement is not limited to oral and written language production; I consider active reading or listening to any LX within and beyond school as a form of behavioural engagement with languages, as they also reflect ‘actions’ learners take.

Cognitive engagement with languages is seen in this study as encompassing focused attention at, active reflection on, and noticing of languages. Research has identified attention, awareness, and noticing of language features as key cognitive processes for language development (Robinson et al., 2012). In this study, however, cognition is not narrowed to language form. From a holistic perspective, cognitive engagement with language is seen here as comprising conscious attention to all aspects of the LX learners encounter in their lives, including communicative and pragmatic aspects of language use.

Finally, emotional engagement with languages is seen as encompassing positive and negative affective reactions and attitudes to LX, including, among others, enjoyment, interest, and boredom. Research has shown the vital role played by positive and negative emotions for language learning (e.g. MacIntyre & Vincze, 2017). While a growing number of recent studies have broadly explored learners’ attitudes in multilingual contexts (e.g. Dekker et al., 2021), there is a critical lack of research on how learners engage affectively with the different LX they encounter within and outside school in their multilingual lives (as an exception, see Ross & Rivers, 2018).

## II Aim of the study

This study explores the ways in which multilingual learners engage with their whole language repertoires within and beyond the English classroom. The study draws on the construct of ‘engagement with LX’ to examine how MS learners relate to, employ, and reflect upon the multiple LX they encounter across the different contexts of their lives. By gaining a deeper appreciation of the multilingual realities of learners within and outside of school, the study seeks to understand how language skills, knowledge, and resources acquired beyond the school context can be transferred to the English classroom. Furthermore, by looking at behavioural, cognitive, and affective dimensions of engagement with LX, the study aims to broaden the theoretical understanding of this construct in its multifaceted complexity. The study is guided by the following exploratory research questions:

• Research question 1: What characterizes the behavioural, cognitive, and affective engagement with LX of MS learners in the English classroom?
• Research question 2: What characterizes the behavioural, cognitive, and affective engagement with LX of MS learners beyond the English classroom?

## III Methods

This study is part of a broader funded project addressing three levels of engagement: engagement with tasks in the English language lesson, engagement with school, and engagement with LX. This study focuses on engagement with LX within and beyond the English language classroom.

### 1 Participants

The data for this study were collected from a first-year English class (*n* = 14) in a MS situated in a suburban area of the Styria region of Austria. Focal students were nine learners who volunteered to participate in the study and provided consent to be video- and audio-recorded during the lesson. This class and school were chosen due to the variety of students’ linguistic, cultural, and socio-economic backgrounds and willingness of the class teacher to allow access to her students. Participating students’ LX repertoires were varied; four students had one or more additional LX alongside German and English, while the remaining five students’ LX were German and English. Students’ average English proficiency level corresponded to the A2 level of the Common European Framework of Reference (CEFR). All students were taught by the same teacher, who had 4 years of teaching experience in MS. Participants’ biographic details are shown in Table 1. Pseudonyms are used to conceal students’ identities.

**Table 1.** Participants’ biographic details.

Name	Age (years)	Gender	LX spoken at home	Other LX
Leonie	12	female	German	
Niko	n/a	male	German; Slovenian; Albanian	Croatian; Bosnian
Luka	13	male	German; Slovenian; Albanian	Serbian
David	12	male	German; Spanish	
Yaran	n/a	male	Kurdish	German; Turkish
Ingrid	13	female	German	
Petra	12	female	German; English	
Susanne	12	female	German; English	
Emma	12	female	German	

*Note*. Pseudonyms are used to conceal students’ identities.

### 2 Instruments and procedures

Data for this study were collected using a biodata online questionnaire, classroom observations, video-audio recording of the lesson, and semi-structured interviews.

Before the start of the data collection procedures, participating students took part in an online survey aimed at gathering background information about their age, gender, languages spoken at home, and any other additional language they used in their lives within and beyond school. The purpose of these data was to contextualize learners’ interview responses and to gain insight into participants’ demographics.

One naturally occurring English lesson taking place in January 2023 was observed and video-recorded by the project leader and two research assistants. The lesson included a variety of tasks including discussion tasks, a grammar task which learners completed on their laptop, and reading tasks. Classroom observation notes were taken by one member of the research team using a structured observation protocol with time indications. Data from the observation protocol enabled the comparison of learners’ interview responses with their observed classroom behaviour and interactions (Fredricks & McColskey, 2012). The observation protocol focused on indicators of behavioural, cognitive, and emotional engagement with LX during the lesson (e.g. initiating interaction with peers and/or the teacher using LX; discussing and/or reflecting about language-related issues). For triangulation purposes, another member of the research team, sat in a different part of the classroom, took detailed unstructured observation notes with time indications, with a particular focus on contextual factors and classroom events that could impact on learners’ engagement during the lesson.

Video and audio data from the lesson were collected to complement the observation notes with details that may be overlooked by the two observers. Two camcorders were placed in an unobtrusive position in two different corners of the classroom, and an audio-recorder was placed on each desk to capture students’ interaction while involved in group work. The video- and audio-recordings enabled a grounded understanding of the classroom dynamics and discourse (Sulis & Philp, 2021). Furthermore, they provided data regarding learners’ LX use during the lesson, including translanguaging practices; these data were later compared with their interview data.

Immediately after the lesson, individual post-lesson interviews were conducted by two members of the research team with the nine students. The interviews were based on a semi-structured interview protocol which was organized around five main sections. The first section aimed at familiarizing with learners, and exploring their self-concept and sense of efficacy in relation to the English class and the English language more broadly (e.g. ‘How do you feel about your progress with English?’). The second part of the interview, for the purpose of the broader research project, drew on stimulated recall procedures based on the video-recording of the lesson, and addressed behavioural, cognitive, and emotional engagement with tasks in the English lesson (e.g. ‘Overall, to what extent did you participate in today’s lesson? Were there any activities where you felt more/less involved?’). The third section of the interview concerned learners’ behavioural, cognitive, and emotional engagement with all the LX in their language repertoire (e.g. ‘Over the past week, have you used English outside the classroom? If so, in which situations?’). The final part of the interview aimed at examining learners’ engagement with school and their broader ecology (e.g. ‘How would you describe your relationship with your school peers?’). Interviews were conducted in German, given that all students in the classroom were highly proficient and reported feeling at ease with using this language. Each interview lasted approximately 20 minutes.

### 3 Ethics

Before the start of the study, ethics approval was obtained by the Ethics Commission of the University of Graz. Prior to data collection, teachers, students, and their legal representatives were asked to carefully read a participant information sheet which informed them about the study, their role in the project, their options for withdrawal, and the measures taken to collect, store, and disseminate the data. Given that the participating students were minors, approval was sought from the English teacher, students themselves, and students’ legal representatives, who were asked to sign a consent form. Students and their legal representatives were given two different participation options: (a) full participation (including face and voice), (b) participation with audio only. Participants who did not give consent to be filmed sat outside the camera range. All data were collected in the school context during school time. Participation was entirely voluntary, and students and their legal representatives were given the option to withdraw from the study at any point up to the publication of findings. Upon transcription, all data were anonymized, and any personal information from the participants’ contribution was removed. Pseudonyms were used at all stage of the research to protect the identity of the participants and conceal their original names and name of the school.

### 4 Analysis

In order to provide an ecologically valid account of the teacher and students’ behaviour, classroom activities, and any other critical events occurring during the lesson under investigation, the classroom observation notes and observation protocol were digitalized and collated with audio and video-data from the lesson. A log with time indications was created with these data.

The interview data were transcribed verbatim and translated into English by a research assistant, and subsequently double checked by a third member of the research team to ensure accuracy of the translation. The choice of English was due to the author’s familiarity with this language and ease of analysis; however, potential issues and implications in translating such data were taken into consideration. Translation is ‘essentially a boundary crossing between two cultures’ (Halai, 2007, p. 345), which suggests that meaning and cultural nuances can be lost in translation, thus potentially affecting the validity of the data. While acknowledging these potential biases, I hope that the translation of the interview data conducted for this study can do justice to the participating students’ voices and perspectives.

The initial process of analysis involved becoming familiar with the data by reading the transcript multiple times and adding extensive memos. This process was followed by a first round of inductive, line-by-line coding using Atlas.ti, taking a Grounded Theory-informed approach (Charmaz, 2006). After the first round of inductive coding, the initial coding list was extensively discussed and refined with a project collaborator. A second wave of coding was then conducted using the refined code list, which included 57 codes. During this stage, all quotes falling within each code were examined in detail, and broader categories and subcategories guided by the research questions of the study were generated (e.g. behavioural engagement with LX during the lesson).

In the final step of analysis, data from the interviews and lesson log were triangulated, in order to obtain a holistic account and contextualized understanding of learners’ engagement with LX, including English. This enabled a cross-comparison of learners’ observable engagement, contextual factors, and salient events occurring during the lesson with their own accounts and perspectives, thus providing a comprehensive picture of learners’ engagement with LX.

## IV Findings

This section presents the findings of the study. It is organized around two main foci, addressing engagement with LX in the English language classroom and beyond the language classroom. The interconnections between the two settings are highlighted throughout.

### 1 Engagement with LX in the English language classroom

All nine participants engaged with LX in distinct ways in the English classroom. All students reported on their active involvement with and use of English language during the lesson; however, the quality and amount of such behavioural engagement differed across participants, depending on a range of intra-individual and contextual factors. Only one participant, Leonie, reported having used English continuously during the lesson: ‘I always stayed with English’. In particular, she highlighted the importance of being exposed to and actively using the language for learning, which may be one of the reasons underlying her persistence with using English during the lesson: ‘I think it’s good that she [the teacher] talks to us in English in English class and that we all speak English, because that’s how we learn it.’

The remaining eight students reported continuously switching between German and English during the lesson, especially when they encountered challenges in finding the right words or structures to express themselves, or in-between tasks to chat with their peers. This was the case, for instance, of Petra; she reported using overall more German during the lesson, and added: ‘But we also talk in “Denglish”: German and English.’ However, when asked about her engagement in a discussion task, she mentioned: ‘We speak in English for that kind of task. Unless we don’t know the word, then we say it to each other in German.’ Indeed, observation and video-audio data from the lesson confirms that she mainly used English during the discussion task, and only switched to German when unsure about vocabulary. This shows that the type of task and opportunities for authentic language use provided by the task itself appeared to have had an impact on her behavioural engagement with English during the lesson. Likewise, two other students reported using English only when prompted to by the task itself; an example was provided by Niko: ‘I used German more than English [. . .] but when it was compulsory, I spoke English.’ Interestingly, one student, Yaran, felt that he could not use any languages other than English in the English class: ‘There is no other language except English that I can use in English class.’ However, as corroborated by the observation data, he also spoke German to his peers, and especially one peer who struggled with English, in order to favour communication: ‘[I speak] German with my friends. Because the one in the middle has a lot of issues with English. So it was better for me to speak German with him.’ This highlights the social and communicative dimensions of alternating between languages in the classroom setting.

While all students switched between English and German during the lesson, Luka reported also using Slovenian, one of his five LX, to communicate with his peer; he provided an interesting account of how he used his whole language repertoire during the English lesson: ‘I spoke Slovenian with my peer. [. . .] And in my mind I was thinking in Albanian [. . .] And I spoke German and English out loud.’ This example reveals Luka’s awareness about how the different languages in his repertoire were used throughout the lesson and the different purposes they served. Indeed, when we also examine the cognitive dimension of learners’ engagement with LX alongside the behavioural one, the picture becomes much more complex. Luka further explained that, at some point during the lesson, he reflected about all the languages in his repertoire: ‘[I thought] about all of them. All of them at the same time.’ This meta-linguistics awareness and active reflection about languages was perhaps one of the reasons underlying his success in the English class, despite the minimum effort invested into studying for tests and exams: ‘I just pay a lot of attention. Like this year, I just got a B in English. I don’t know how I did it – I barely studied for the exam.’ Similarly, when asked about his effort in learning other subjects, he explained: ‘In German, so-so. But I’ve improved. That’s why everything works out perfectly now.’ As will be seen in the following section, Luka showed an intense engagement with LX beyond the classroom, which appeared to have positively contributed to his achievement in the English and German classes.

Another example of cognitive engagement with different LX during the lesson was provided by Yaran; with reference to the final discussion task, he reported: ‘I thought about it a bit in Turkish. Just for fun.’ Interestingly, however, when asked about the connections he made between the different languages in his repertoire during the lesson, he replied: ‘I only make connections like that between Turkish and Kurdish and with the two I also do – like, only make connections between English and German.’ Like Luka, he reported not investing a great effort into studying English; however, he mentioned an overall improvement: ‘My English is getting better. That’s what’s so great about it.’ Finally, David reported making connections between English and German throughout the lesson, but not between English and Spanish, one of his LX: ‘I think about German, but not about Spanish.’ This may be due to the fact that learners typically tend to make more connections between closely related languages (Festman, 2021), which is the case of German and English. Another reason may be that German is the main language of instruction in the school context and, given the continuous exposure to this language, learners may make connections to German with more ease compared to other LX in their repertoire.

As for the five students with only German and English in their repertoire, they did not seem to show the same level of metalinguistic awareness as their highly multilingual peers. However, Leonie’s excerpt shows that, the more learners are prompted to reflect about the language itself, the more they can understand its inner workings: ‘At first, with grammar, I don’t understand it. But then the more we do it, the more I start to understand it.’ This shows that a balanced and synergetic approach including both implicit and explicit linguistic instruction can benefit learners’ noticing and metalinguistic awareness (see also R. DeKeyser, 2009), especially when students are not accustomed to actively reflect about languages.

In terms of emotional engagement with LX during the lesson, it is interesting to note that three of the highly multilingual students reported feeling generally neutral about this language. When Niko was asked about his feelings while using English to communicate, he replied that it felt ‘normal’. Similarly, Yaran’s feelings towards English were reported as being ‘just neutral’; however, he also mentioned that he had fun using English during the reading tasks, suggesting that the type of task at hand can influence learners’ emotional engagement with LX. In contrast, the five students with only German and English in their repertoire reported on their enjoyment of English during the lesson, showing overall positive engagement with this language.

### 2 Engagement with LX beyond the lesson

Learners’ engagement with LX beyond the lesson also appeared to follow unique trajectories depending on a complex interplay of intra-individual and socio-contextual factors. A key determinant of the quality of students’ engagement with LX concerned the specific language at hand. All learners reported using English beyond the classroom, albeit in different ways and with different purposes. Five students reported watching English language films and videos in their free time. Luka, for instance, highlighted the benefits of watching films for language learning: ‘I find that, if I watch films in English or something, I can learn more. I mean, I’ve learned more from watching movies than in class’. Another instance was reported by Yaran, who attributed his English progress more so to watching films and videos than the English lessons: ‘My English is getting better. That’s what’s so great about it. Other than that – my English language comes more from watching movies and stuff.’ A similar account was provided by Leonie: ‘Personally, I think I learn more English – I rather learn to speak English from English videos and stuff. Like, how to pronounce the words, I learn that more from English songs or videos. But I also learn English in class.’ This suggests that these learners were not just passively learning English through watching films and videos but, to some degree, they were also cognitively engaged with this language, through paying attention to specific language features such as pronunciation or vocabulary. Similarly, four learners reported reusing some of the vocabulary and structures learnt through playing video-games and browsing social media in the English class. An example was provided by Petra: ‘For example, we had the word “exam” [. . .] I heard it a lot on TikTok, so I knew it, and then I used it a lot.’ Earlier research has also shown the benefits of exposure to video-audio elements for learners’ vocabulary (e.g. Webb & Rodgers, 2009) and oral skills (e.g. Dikilitas & Duvenci, 2009).

Notably, the examples shown earlier also revealed that some of the participants perceived audio-visual media as a more important contributor towards their English learning than formal instruction. Data collected in the Norwegian context have also shown that learners perceive English language media and popular culture as a more important sources of English knowledge compared to school instruction (Busby, 2015). Naturally, this poses a risk for English teachers’ professional standing and suggests that English as a school subject needs to be reflected upon possibly to incorporate other competences and skills alongside those typically taught in the English class to maintain its relevance (Haukås, Mercer, & Svalberg, 2022). An exception to this pattern, however, was provided by Petra. Unlike her peers, she felt that she could learn more in her English lessons than beyond the classroom: ‘But I take more from the lessons.’ This shows a certain degree of individual variation in terms of learners’ perceptions of their sources of English knowledge.

Finally, learners also reported using English to communicate with parents (*n* = 2), siblings (*n* = 4), friends (*n* = 3), and when going abroad (*n* = 2). Susanne, for instance, used a mix of English and German to communicate with her family: ‘I talk to my parents in a mixture of English and German. [. . .] With my siblings, too.’ Another example was provided from Petra; she reported frequently practicing English with her family and friends, not only to improve her skills, but also because she found it inherently fun, thus suggesting positive emotional engagement with this language: ‘I sometimes speak English for fun at home with my mum and with friends [. . .] Neither of us can do it well, but if something is wrong, then we try to get it right.’ The remaining students, however, reported that their parents did not reach a sufficient level of English proficiency to support them with their English learning.

Just like Petra, four other students reported on their positive emotional engagement with the English language. Ingrid mentioned: ‘Well, I like English’. She continued: ‘It’s fun when you know that you speak another language. For example, on holiday my whole family couldn’t speak English except for me. That was cool.’ Leonie also reported on her positive emotional engagement with the English language; however, unlike Ingrid, she revealed a more negative attitude towards multilingualism: ‘I don’t want to learn too many languages, because then my German might get worse [. . .] or that I might speak the other language more than my real mother tongue.’ This suggests that, in some contexts, there is still the misconception that knowing more than one language may hinder one’s first language; as such, raising learners’ awareness regarding the benefits of multilingualism should become a priority.

Learners’ engagement with the other LX in their repertoires, and how these interrelated with English, were determined by their unique language learning histories. David, for instance, spoke a mixture of German and Spanish at home. When asked about how he learnt this language, he replied: ‘I come from Spain,’ thus suggesting that being Spanish was part of his sense of identity. Although Spanish was his L1, however, he reported that German was the easiest LX for him, and that he struggled with written Spanish: ‘There are some words I don’t know how to spell.’ Furthermore, as seen earlier, he tended to make connections between German and English, but not between Spanish and English. This suggests that, drawing on the traditional dichotomy between L1 and other LX may not always provide an accurate picture of learners’ multilingual lives; especially when considering individuals with a migration background, looking at their language repertoire holistically can provide a more nuanced account of their engagement with LX.

A different language learning history was the one narrated by Yaran, whose LX were Kurdish and Turkish alongside German and English. He reported that his family’s language was Kurdish, and further explained: ‘I am a Kurd from Turkey. As a Kurd I don’t really have a country, so my home country – my citizenship – is Turkey, and because I come from Turkey, I also speak that language.’ Yaran’s words suggest the close interconnection between language and national identity. He also reported engaging with the Turkish language to communicate with his uncle from time to time: ‘For example, me and my uncle, we mainly speak Kurdish, and then I said to him, we should use Turkish – just to use something new. And when we started, it just sounded – it felt like I was talking to someone else [. . .] It’s a really special feeling.’ As for German, he reported using this language to communicate with his siblings and as the main language of instruction at school. However, he found the German grammar particularly complex, which led to frustration, suggesting negative emotional engagement with this language: ‘It’s just, there’s this dative and all this stuff [. . .] I find it unnecessary and difficult.’ Nonetheless, he invested a substantial amount of attention in the German class: ‘In German, I try to pay as much attention as possible.’

Niko’s engagement with LX beyond the classroom appeared yet very different. He learnt Slovenian and Albanian through his family; as such, these two LX appeared to play a key role in Niko’s language repertoire. When reflecting on these languages, he reported on the complexity of regional varieties, which served as a source of struggle for him: ‘There is also High Slovenian, just like High German. [. . .] It’s quite difficult for me sometimes, because when I talk, it [my throat] always hurts so much. I’m from around [anonymized] [. . .] and it’s just a bit different there.’ Niko’s attention to dialectal varieties suggests a certain degree of noticing and reflection about languages. Furthermore, he also reported having autonomously acquired two other Slavic languages, Croatian and Bosnian: ‘I know a few other languages as well, but I taught those to myself, I guess.’ He described how reflecting on the LX input he received helped him with language production: ‘Once you hear it, you can sort of think of how to say it.’ Interestingly, he also reported being able to understand English quite easily, suggesting that the process of noticing and reflecting about language issues also occurred with English: ‘I already understand everything.’

Finally, Luka’s LX repertoire also included several Slavic languages, alongside English and German. He mainly used Albanian and Slovenian at home; however, he felt that he had learnt these languages on his own, rather than through his family: ‘They didn’t teach me. I wanted to try it on my own, so I tried it.’ He further explained: ‘That’s how I taught myself, just by speaking it.’ Furthermore, he reported having learnt Serbian on his own to communicate with his Serbian friends; he explained the similarities between Serbian and Slovenian: ‘It’s actually the same as Slovenian, but it’s just different words, and that’s why it’s more difficult.’ He further mentioned that his friends would understand Slovenian, but he still tried to speak Serbian with them: ‘Most of the time if I say it in Slovenian, they understand it, anyway. [. . .] But I speak Serbian with them.’

In sum, the findings suggest a very complex picture of engagement with LX beyond the classroom, depending on a complex web of factors including learners’ biographies, family histories, previous language knowledge, interests and motivation, as well as affordances for language learning beyond the classroom.

## V Discussion and implications

The purpose of this study was to explore learners’ engagement with LX within and beyond the English classroom. The discussion of the findings and related implications will focus on three main interrelated areas: the multilingual nature of the English language classroom, multilingual realities beyond the classroom, and a holistic perspective on learners’ multilingual lives.

### 1 The multilingual English language classroom

One key finding of the study concerns the multilingual nature of the English language classroom. At first glance, learners only seemed to engage with English and German throughout the lesson, and switched between these languages when required by the task, the teacher, or a specific communicative purpose. As shown by the findings, learners experienced higher behavioural engagement with English during activities that promoted authentic language use and where using English felt necessary to achieve the task purpose (e.g. discussion activities in pairs or small groups). These findings align with previous research; Lambert and Zhang (2019) explain that, ‘situationally authentic communication involves learners personally and affectively as well as engaging them cognitively in L2 processing and the negotiation of meaning’ (pp. 391–392). Research has also revealed that tasks requiring learners to generate their own content (e.g. discussion tasks) can be more behaviourally, cognitive, and emotionally engaging compared to tasks with teacher-generated content (Lambert et al., 2017).

In contrast, participants tended to use more German when they felt they did not possess the vocabulary to express themselves with peers or the teacher, ask for clarifications, or chat between tasks. In a similar vein, a review on L1 use in foreign language classrooms by Shin et al. (2020) highlighted the relational nature of L1 use in L2 learning context, and underscored its importance during classroom and group talk. While, for several students in this study, German was not their L1 but served as one of the multiple LX in their repertoires, it was nonetheless employed as a lingua franca in the English classroom and at school more broadly.

Yet, many more languages were present in the classroom than seemingly drawn on. Niko, for instance, employed Slovenian, one of his LX, to interact with one of his peers during the lesson, and alternated between English and German to communicate with the teacher and to complete the tasks, suggesting that he drew on his rich multilingual repertoire to cope with different situations. Typically, multilingual children develop the ability to distinguish between languages and adapt to different interlocutors and contexts from a young age (Ticheloven et al., 2021). However, in the case of our participants, these translanguaging practices were not always verbalized; as seen earlier, while involved in the different tasks, learners mentally engaged with their different LX in their repertoire during the lesson, but mainly expressed themselves audibly in German and English. This suggests that, while engagement with different LX during the English lesson may not be visible to teachers or peers, it does not mean it is not happening. Indeed, even when students are silent, they may be cognitively engaged with their different LX; as explained by García (2020), ‘when silence is active, it gives way to inner speech,’ which is central to translanguaging practices (see also García & Wei, 2014).

As pointed by the findings, raising learners’ and teachers’ awareness of the multilingual potential in the classroom should be prioritized. First, raising such awareness would enable learners to maximize the positive effects of multilingualism on additional language learning. A plethora of studies have shown that language mixing does not hinder learning (see, for example, Antón et al., 2016); in contrast, multilingual students were found to possess an advantage when acquiring additional languages (Cenoz, 2003), particularly in terms of metalinguistic awareness (e.g. Jessner, 2008; Rauch et al., 2012). This was also shown in these data; students with three or more LX in their repertoire displayed higher metalinguistic awareness compared to their peers who only had German and English in their repertoire. However, it is also important to note that, whether these advantages translate into higher academic achievement and linguistic development is also contingent upon factors such as the status of students’ LX within the education system and society (Cenoz, 2003), their socio-economic status (OECD, 2015), and proficiency in the majority language (Genesee et al., 2006). While some of these factors are beyond teachers’ control, teachers can take action to promote multilingualism in the English classroom, with gains for all students’ engagement with LX. For instance, teachers can enhance learners’ cognitive engagement with LX by prompting students to actively reflect on the different languages in their repertoire and consider the similarities or differences between these languages and English (see, for example, Cenoz & Gorter, 2011). This can maximize learners’ pre-existent metalinguistic awareness, thereby enabling learners not only to gain a deeper understanding of the inner workings of these languages, but also to transfer their language skills to any additional LX (Hofer & Jessner, 2019; Jessner, 2018).

Second, by recognizing the multilingualism present in the English language classroom and appreciating the linguistic resources of all students, teachers can boost their self-confidence, feeling of belonging, and motivation (Herzog-Punzenberger et al., 2017). As explained by Haukås, Storto, and Tiurikova (2022), ‘for students in school, knowledge of potential benefits of being multilingual may be an important trigger for them to decide to and continue learning languages’ (p. 2); as such, teachers could explicitly discuss the benefits of multilingualism with students. Furthermore, reflecting on the different LX present in the language classroom can also enhance learners’ understanding of linguistic diversity and foster their intercultural competences (Eren, 2022; Kalaja & Pitkänen-Huhta, 2020). As highlighted by García (2017), students ‘rarely acquire awareness of the rich multilingualism in their classroom, beyond the languages sanctioned by schools’ (p. 268). Learning from one another’s linguistic and cultural backgrounds can trigger learners’ curiosity towards other LX and related cultures (Hedman & Fisher, 2022), thereby increasing their emotional engagement with LX. Furthermore, it can promote the development of intercultural competences, empathy, tolerance, and compassion among learners (Driscoll, 2017), and foster a sense of inclusivity in the classroom.

### 2 Multilingual realities beyond the classroom

The findings suggest that learners’ behavioural, cognitive, and emotional engagement with LX beyond the classroom followed unique trajectories influenced by individual and socio-contextual factors. Among the intra-personal factors shaping their engagement with the LX in their repertoires, learners’ biographies, family histories, previous language knowledge, interests, and motivation seemed to play a critical role. In the case of Yaran and David, for instance, engagement with the LX in their repertoires appeared to be intertwined with their sense of national identity. Language, indeed, serves as ‘a crucial tool to be able to become part of and identify with the nation’ (G.N. Dekeyser et al., 2016, p. 2). It has a symbolic function as it embodies ‘our attachments, our sense of our selves, what kinds of belonging we wish to invoke and display’ (Lo Bianco, 2010, p. 24). Furthermore, the findings highlight the influence of social relationships on learners’ engagement with LX beyond the classroom; learners reported switching between their different LX, including English, to interact with family members and peers, and during trips abroad, revealing the inherently social dimension of engagement with LX. This supports the idea that, ‘multilingual interaction takes place at different societal levels, in different social spaces, and with very different interlocutors’ (García, 2018, p. 882).

In addition to switching between languages depending on the interlocutor and context, our participants with three or more LX in their repertoire drew on languages already known to learn new ones, thereby suggesting a ‘catalytic effect’ (Festman, 2021). This was especially the case of LX belonging to the same language families; naturally, ‘closely related languages might be easier to learn than others’ (Antoniou et al., 2015, p. 684), due to the similarities between such languages and the relative ease of positive transfer. A notable example is that of Luka, who learnt Serbian to communicate with his friends, and Niko, who learnt Croatian and Bosnian. This suggests learners’ intense cognitive engagement with their different LX, as they made use their pre-existent linguistic resources, while also processing the new linguistic input they were exposed to. In order to connect learners’ engagement with LX beyond the classroom to their English learning in the classroom, teachers could embrace this ‘catalytic effect’. For instance, they could ask learners to reflect on the similarities and differences between the other LX in their repertoires and English, to increase their metalinguistic awareness and create opportunities for noticing (see, for example, Cenoz & Gorter, 2014; Cenoz & Santos, 2020). Furthermore, as also seen earlier, teachers can connect learners’ diverse LX to English in the classroom by using the English language classroom as a space where learners can share and discuss their different LX and related cultures (Babic et al., 2022; Driscoll, 2017). The findings suggest indeed a need to rethink English as a school subject and thus incorporating intercultural competences and global skills in the English curriculum would contribute to enhancing the relevance of English as a school subject (Babic et al., 2022; Haukås, Mercer, & Svalberg, 2022).

Another key finding of the study concerns the unique nature of engagement with the English language, which appeared to differ from learners’ engagement with the other LX in their repertoires. Such distinction may be due to the ubiquity of the English language across social media, films, music, and video games, which provides unique affordances for learners (Brevik, 2019; Lee, 2023). Participants reported engaging with English in various ways beyond the classroom; for instance, through watching films, playing video games, and using social media. These media were perceived by participants as significant contributors towards their English learning, in some cases, even more so than formal classroom instruction. These findings suggest that learners’ engagement with the English language beyond the classroom entailed much more than passive exposure to this language. Rather, it involved actively processing multiple language features such as vocabulary and pronunciation, and reusing what they learnt beyond the classroom in the English lesson, thereby indicating a complex blend of behavioural and cognitive engagement with English. Affordances for language learning outside the classroom can provide indeed valuable opportunities for interactive, social, and multimodal learning (Jones & Hafner, 2021), and for the acquisition of vocabulary that is not typically included in the regular language class (Richards, 2015).

Language educators should thus encourage and support learners to engage with English outside the classroom, but also create a bridge between the two contexts (Chern & Dooley, 2014; Reinders & Benson, 2017). In addition to developing classroom material and activities that foster authentic language use, they could also create explicit connections to students’ engagement with English beyond the classroom (Benson, 2011). For instance, teachers could ask students to comment on or give presentations on English language books, films, social media videos, music, or any other media of their choice; by drawing on their interests beyond the classroom, teachers can support learners’ emotional engagement with English. As highlighted by Schwienhorst (2009), some affordances need teachers’ support to be recognized; as such, teachers can suggest themselves different ways in which learners can engage with languages beyond the classroom, by providing recommendations about books, films, web pages, or any other media that may be of interest to their students. Finally, teachers can also provide learners with practical strategies on how to capitalize on the affordances they perceive outside the classroom (Tweed & Reinders, 2023), such as, for instance, by taking note of the new vocabulary or structures they encounter, and giving them the opportunity to ask questions and discuss about these in the classroom.

### 3 A holistic perspective on learners’ multilingual lives

A key lesson learnt from the study concerns the need to take a holistic perspective in relation to learners’ engagement with LX. In particular, the study suggests two distinct, yet interrelated ways to consider learners’ multilingual lives from a holistic perspective.

The first way to consider learners’ engagement with LX holistically is by looking at learners’ whole LX repertoire to understand how they engage with the different LX they encounter at home, at school, and beyond. This approach can provide a more nuanced picture of learners’ engagement with the different LX in their lives that goes beyond the school setting and isolated tasks. At present, the limited available research on engagement with language has focused on second language learning (e.g. Ahn, 2016; Baralt et al., 2016). However, as shown by this study, learners possess a complex repertoire of languages, which they used cross-linguistically rather than in isolation (see also Cenoz & Gorter, 2011), within and across their different areas of life. Therefore, a holistic approach can help establish ‘bridges between second/foreign language teaching at school and multilingualism in real-life communication’ (Cenoz & Gorter, 2015, p. 8). Another important aspect to consider is that engagement with language is usually examined in the context of language related episodes (LREs) or collaborative dialogue (Svalberg, 2009) either in laboratory settings or in the formal instruction context, thus focusing on timescales that typically do not exceed more than a matter of minutes. However, as revealed by the findings, engagement with LX can be understood as a dynamic process occurring across multiple contexts and multiple timescales, potentially ranging from one short instructional episode to one’s lifetime. Furthermore, earlier studies have researched engagement with language mostly in the context of learners’ observable behaviour and interactions (as an exception, see Baralt et al., 2016); in contrast, a holistic perspective examines observable and unobservable determinants of learners’ behavioural, cognitive, and emotional engagement with LX, as well as objective and subjective experiences of engagement.

The study has also revealed the criticality of looking at the learner and the LX not as separate and isolated entities, but as a whole. The findings have shown that learners’ multiple LX are integral part of their identities and lives more broadly, thus highlighting the interconnectedness between the learner and the LX. A holistic perspective on engagement with LX does not separate the agent (i.e. the learner) from the object (i.e. the language), as in the typical conceptualization of the construct (Svalberg, 2009), but it recognizes the LX in the learners’ repertoire as an integral part of the learners’ self. As highlighted by Norton (2016), ‘language is not only a linguistic system of words and sentences, but also a social practice in which identities and desires are negotiated in the context of complex and often unequal social relations’ (p. 476). As such, when exploring learners’ engagement with LX, we cannot leave out of consideration their unique biographies, identities, and the complex social contexts in which they are embedded.

## VI Conclusions

The purpose of this study was to explore learners’ engagement with LX to gain insight into how learners reflect on, relate to, and use their whole language repertoire within and beyond the English language classroom. The study drew on and redefined the original construct of engagement with language (Svalberg, 2009), by holistically addressing learners’ engagement with all the LX in their repertoire across the different contexts of their lives.

The study has several limitations. First, given the specific context where the research project was conducted, namely, a suburban middle school in Austria, the findings cannot be generalized to other contexts. Furthermore, the sample of the study was relatively small; including several classes in different schools would have certainly provided a richer picture of learners’ engagement with LX. The study also drew on data collected at one point in time; collecting data at multiple time points could have provided further insights into the dynamism of the engagement with LX. Furthermore, the presence of the researchers in class may have compromised the authenticity of the classroom atmosphere and dynamics. Nonetheless, it is hoped that the insights gained from the study could be valuable to broaden knowledge of this construct and suggest avenues for further research. For example, as suggested earlier, further research on engagement with LX could take a dynamic perspective and focus on different timescales, to understand how it changes over time. Additionally, research on LX is needed across a variety of contexts, from primary education to adult learning, to better understand how it can be fostered depending on students’ diverse needs, affordances, and motivations. Furthermore, further research could examine the relationship between engagement with LX and other types of engagement (e.g. engagement with school; engagement with tasks), to shed light on the unique characteristics of this construct and understand its interconnections with other facets of engagement. Finally, this study only touched the surface of the relationship between learners’ engagement with LX and intercultural competences; further studies are needed to examine their connections.

Through holistically exploring learners’ engagement with all the LX they come into contact across the different domains of their lives, the study has shed light on learners’ multilingual lives within and beyond the classroom and has revealed the interconnections between these domains. The study has also furthered understanding of the ways learners’ engagement with LX beyond the classroom can support their learning in the English language classroom, and the kind of affordances learners perceive in their multiple contexts to enhance their learning process. Finally, the findings have also led to the suggestion of practical implications in terms of how teachers can engage with learners’ whole LX repertoire to support their language learning process, while fostering a sense of inclusivity in the classroom and promoting learners’ intercultural competences.

## References

[bibr1-13621688231216869] Ahn S.Y. (2016). Exploring language awareness through students’ engagement in language play. Language Awareness, 25, 40–54.

[bibr2-13621688231216869] Antón E. Thierry G. Goborov A. Anasagasti J. Duñabeitia J.A. (2016). Testing bilingual educational methods: A plea to end the language-mixing taboo. Language Learning, 66, 29–50.

[bibr3-13621688231216869] Antoniou M. Liang E. Ettlinger M. Wong P.C. (2015). The bilingual advantage in phonetic learning. Bilingualism: Language and Cognition, 18, 683–695.

[bibr4-13621688231216869] Babic S. Platzer K. Gruber J. Mercer S. (2022). Positive language education: Teaching beyond language. Humanising Language Teaching, 24, 1–9.

[bibr5-13621688231216869] Baralt M. Gurzynski-Weiss L. Kim Y. (2016). Engagement with language: How examining learners’ affective and social engagement explains successful learner-generated attention to form. In Sato M. Ballinger S. (Eds.), Peer interaction and second language learning: Pedagogical potential and research agenda (pp. 209–240). John Benjamins.

[bibr6-13621688231216869] Benson P. (2011). Language learning and teaching beyond the classroom: An introduction to the field. In Benson P. Reinders H. (Eds.), Beyond the language classroom (pp. 7–17). Palgrave Macmillan.

[bibr7-13621688231216869] BIFIE. (2020). Standardüberprüfung 2019: Englisch, 8. Schulstufe Bundesergebnisbericht [Standard examination 2019: English, 8th grade federal results report]. BIFIE. Available at: https://www.iqs.gv.at/_Resources/Persistent/fcdf653003a5d1a4755fdc823749a75360b2f30f/BiSt-UE_E8_2019_Landesergebnisbericht_Salzburg.pdf (accessed November 2023).

[bibr8-13621688231216869] Brevik L.M. (2019). Gamers, surfers, social media users: Unpacking the role of interest in English. Journal of Computer Assisted Learning, 35, 595–606.

[bibr9-13621688231216869] Bundesministerium für Bildung, Wissenschaft, und Forschung. (2020). Mittelschule *[Middle school]*. Bundesministerium für Bildung, Wissenschaft, und Forschung. Available at: https://www.bmbwf.gv.at/Themen/schule/schulsystem/sa/ms.html (accessed November 2023).

[bibr10-13621688231216869] Busby N.L. (2015). Too cool for school? Sources of English language acquisition, attitudes, and academic reading ability among Norwegian university students. Unpublished master’s thesis, NTNU, Trondheim, Norway.

[bibr11-13621688231216869] Cenoz J. (2003). The additive effect of bilingualism on third language acquisition: A review. International Journal of Bilingualism, 7, 71–87.

[bibr12-13621688231216869] Cenoz J. Gorter D. (2011). Focus on multilingualism: A study of trilingual writing. The Modern Language Journal, 95, 356–369.

[bibr13-13621688231216869] Cenoz J. Gorter D. (2014). Focus on multilingualism as an approach in educational contexts. In Blackledge A. Creese A. (Eds.), Heteroglossia as practice and pedagogy (pp. 239–254). Springer.

[bibr14-13621688231216869] Cenoz J. Gorter D. (Eds.). (2015). Multilingual education. Cambridge University Press.

[bibr15-13621688231216869] Cenoz J. Gorter D. (2017). Minority languages and sustainable translanguaging: Threat or opportunity? Journal of Multilingual and Multicultural Development, 38, 901–912.

[bibr16-13621688231216869] Cenoz J. Santos A. (2020). Implementing pedagogical translanguaging in trilingual schools. System, 92, 102273.

[bibr17-13621688231216869] Charmaz K. (2006). Constructing grounded theory: A practical guide through qualitative analysis. Sage.

[bibr18-13621688231216869] Chern C.L. Dooley K. (2014). Learning English by walking down the street. ELT Journal, 68, 113–123.

[bibr19-13621688231216869] Council of Europe. (2023). Language repertoire. Council of Europe. Available at: https://www.coe.int/en/web/lang-migrants/repertoire-language- (accessed November 2023).

[bibr20-13621688231216869] Dekeyser G.N. Puschmann P. Agirdag O. (2020). Multiple languages, multiple identities? Children’s language characteristics and their ethnic and national identification. Language, Culture and Curriculum, 33, 368–383.

[bibr21-13621688231216869] DeKeyser R. (2009). Cognitive-psychological processes in second language learning. In Long M.H. Doughty C.J. (Eds.), The handbook of language teaching (pp. 119–138). Wiley-Blackwell.

[bibr22-13621688231216869] Dekker S.V. Duarte J. Loerts H. (2021). ‘Who really speaks like that?’: Children’s implicit and explicit attitudes towards multilingual speakers of Dutch. International Journal of Multilingualism, 18, 551–569.

[bibr23-13621688231216869] Dewaele J.-M. (2018). Why the dichotomy ‘L1 versus LX user’ is better than ‘native versus non-native speaker’. Applied Linguistics, 39, 236–240.

[bibr24-13621688231216869] Dikilitas K. Duvenci A. (2009). Using popular movies in teaching oral skill. Procedia – Social and Behavioral Sciences, 1, 168–172.

[bibr25-13621688231216869] Driscoll P. (2017). Considering the complexities of teaching intercultural understanding in foreign languages. In Enever J. Lindgren E. (Eds.), Early language learning: Complexity and mixed methods (pp. 24–40). Multilingual Matters.

[bibr26-13621688231216869] Eder F. Altrichter H. Bacher J. Hofmann F. Weber C. (2015). Evaluation der neuen Mittelschule (NMS): Befunde aus den Anfangskohorten *[Evaluation of the new middle school: Findings from the initial cohorts]*. Research report. Universität Salzburg, Johannes Kepler Universität Linz, Pädagogische Hochschule OÖ. Available at: https://www.plus.ac.at/wp-content/uploads/2021/09/ForschungsberichtNMS.pdf (accessed November 2023).

[bibr27-13621688231216869] Ellis N.C. O’Donnell M.B. Römer U. (2013). Usage-based language: Investigating the latent structures that underpin acquisition. Language Learning, 63, 25–51.

[bibr28-13621688231216869] Eren Ö . (2022). Towards multilingual turn in language classes: Plurilingual awareness as an indicator of intercultural communicative competence. International Journal of Multilingualism. Advance online publication. 10.1080/14790718.2022.2090568

[bibr29-13621688231216869] Erling E.J. Brummer M. Foltz A. (2023). Pockets of possibility: Students of English in diverse, multilingual secondary schools in Austria. Applied Linguistics, 44, 72–102.

[bibr30-13621688231216869] Erling E.J. Radinger S. Foltz A. (2023). Understanding low outcomes in English language education in Austrian middle schools: The role of teachers’ beliefs and practices. Journal of Multilingual and Multicultural Development, 44, 412–428.

[bibr31-13621688231216869] European Commission. (2017). Commission staff working document: Country report Austria 2017. European Commission. Available at: https://eur-lex.europa.eu/legal-content/EN/TXT/PDF/?uri=CELEX:52017SC0085 (accessed November 2023).

[bibr32-13621688231216869] European Commission. (2023). Person with a migratory background: Migration and Home Affairs. European Commission. Available at: https://home-affairs.ec.europa.eu/networks/european-migration-network-emn/emn-asylum-and-migration-glossary/glossary/person-migratory-background_en (accessed November 2023).

[bibr33-13621688231216869] Festman J. (2021). Learning and processing multiple languages: The more the easier? Language Learning, 71, 121–162.

[bibr34-13621688231216869] Fredricks J.A. Filsecker M. Lawson M.A. (2016). Student engagement, context, and adjustment: Addressing definitional, measurement, and methodological issues. Learning and Instruction, 43, 1–4.

[bibr35-13621688231216869] Fredricks J.A. McColskey W. (2012). The measurement of student engagement: A comparative analysis of various methods and student self-report instruments. In Christenson S.L. Reschly A.L. Wylie C. (Eds.), Handbook of research on student engagement (pp. 763–782). Springer.

[bibr36-13621688231216869] García O. (2017). Critical multilingual language awareness and teacher education. In Cenoz J. Gorter D. May S. (Eds.), Encyclopedia of language and education: Multilingualism and language awareness (pp. 263–280). Springer.

[bibr37-13621688231216869] García O. (2018). The multiplicities of multilingual interaction. International Journal of Bilingual Education and Bilingualism, 21, 881–891.

[bibr38-13621688231216869] García O. (2020). Singularity, complexities and contradictions: A commentary about translanguaging, social justice, and education. In Panagiotopoulou J.A. Rosen L. Strzykala J. (Eds.), Inclusion, education and translanguaging: How to promote social justice in (teacher) education? (pp. 11–20). Springer.

[bibr39-13621688231216869] García O. Wei L. (2014). Translanguaging: Language, bilingualism and education (pp. 46–62). Palgrave Macmillan.

[bibr40-13621688231216869] Genesee F. Lindholm-Leary K. Saunders W. Christian D. (2006). Educating English language learners. Cambridge University Press.

[bibr41-13621688231216869] Halai N. (2007). Making use of bilingual interview data: Some experiences from the field. The Qualitative Report, 12, 344–355. Available at: http://ecommons.aku.edu/pakistan_ied_pdck/22 (accessed November 2023).

[bibr42-13621688231216869] Hammarberg B. (2010). The languages of the multilingual: Some conceptual and terminological issues. International Review of Applied Linguistics in Language Teaching, 48, 91–104.

[bibr43-13621688231216869] Haukås Å. Mercer S. Svalberg A.M.L . (2022). School teachers’ perceptions of similarities and differences between teaching English and a non-language subject. TESOL Quarterly, 56, 474–498.

[bibr44-13621688231216869] Haukås Å. Storto A. Tiurikova I . (2022). School students’ beliefs about the benefits of multilingualism. Journal of Multilingual and Multicultural Development. Advance online publication. 10.1080/01434632.2022.2075001

[bibr45-13621688231216869] Hedman C. Fisher L. (2022). Critical multilingual language awareness among migrant students: Cultivating curiosity and a linguistics of participation. Journal of Language, Identity and Education. Advance online publication. 10.1080/15348458.2022.2078722

[bibr46-13621688231216869] Herzog-Punzenberger B. Le Pichon-Vorstman E. Siarova H. (2017). Multilingual education in the light of diversity: Lessons learned, NESET II report. Publications Office of the European Union. Available at: https://nesetweb.eu/wp-content/uploads/2019/06/Multilingualism-Report.pdf (accessed November 2023).

[bibr47-13621688231216869] Herzog-Punzenberger B. Schnell P. (2019). Austria: Equity research between family background, educational system and language policies 1980–2016. In Stevens P. Dworkin G. (Eds.), The Palgrave handbook on race and ethnic inequalities in education (pp. 105–158). 2nd edition. Palgrave MacMillan.

[bibr48-13621688231216869] Hofer B. Jessner U. (2019). Multilingualism at the primary level in South Tyrol: How does multilingual education affect young learners’ metalinguistic awareness and proficiency in L1, L2 and L3? The Language Learning Journal, 47, 76–87.

[bibr49-13621688231216869] Jessner U. (2008). A DST model of multilingualism and the role of metalinguistic awareness. The Modern Language Journal, 92, 270–283.

[bibr50-13621688231216869] Jessner U. (2018). Language awareness in multilingual learning and teaching. In Garrett P. Cots J.M. (Eds.), The Routledge handbook of language awareness (pp. 257–274). Routledge.

[bibr51-13621688231216869] Jones R.H. Hafner C.A. (2021). Understanding digital literacies: A practical introduction. Routledge.

[bibr52-13621688231216869] Kalaja P. Pitkänen-Huhta A. (2020). Raising awareness of multilingualism as lived–in the context of teaching English as a foreign language. Language and Intercultural Communication, 20, 340–355.

[bibr53-13621688231216869] Lambert C. Philp J. Nakamura S. (2017). Learner-generated content and engagement in second language task performance. Language Teaching Research, 21, 665–680.

[bibr54-13621688231216869] Lambert C. Zhang G. (2019). Engagement in the use of English and Chinese as foreign languages: The role of learner-generated content in instructional task design. The Modern Language Journal, 103, 391–411.

[bibr55-13621688231216869] Lee Y.J. (2023). Language learning affordances of Instagram and TikTok. Innovation in Language Learning and Teaching, 17, 408–423.

[bibr56-13621688231216869] Lo Bianco J . (2010). Some ideas about multilingualism and national identity. TESOL in Context, 20, 22–36.

[bibr57-13621688231216869] MacIntyre P.D. Vincze L. (2017). Positive and negative emotions underlie motivation for L2 learning. Studies in Second Language Learning and Teaching, 7, 61–88.

[bibr58-13621688231216869] Mercer S. Talbot K.R. Wang I.K.H. (2021). Fake or real engagement: Looks can be deceiving. In Hiver P. Al-Hoorie A.H. Mercer S. (Eds.), Student engagement in the language classroom (pp. 143–162). Multilingual Matters.

[bibr59-13621688231216869] Norton B. (2016). Identity and language learning: Back to the future. TESOL Quarterly, 50, 475–479.

[bibr60-13621688231216869] OECD. (2015). Education at a glance 2015: OECD indicators. OECD.

[bibr61-13621688231216869] OECD. (2016). OECD reviews of school resources: Austria. OECD. Available at: https://www.oecd-ilibrary.org/sites/9789264256729-5-en/index.html?itemId=/content/component/9789264256729-5-en (accessed November 2023).

[bibr62-13621688231216869] OECD. (2017). Education policy outlook: Austria. OECD. Available at: https://web-archive.oecd.org/2021-01-22/450269-education-policy-outlook-country-profile-austria.pdf

[bibr63-13621688231216869] Otwinowska A. (2017). English teachers’ language awareness: Away with the monolingual bias? Language Awareness, 26, 304–324.

[bibr64-13621688231216869] Philp J. Duchesne S. (2016). Exploring engagement in tasks in the language classroom. Annual Review of Applied Linguistics, 36, 50–72.

[bibr65-13621688231216869] Rauch D.P. Naumann J. Jude N. (2012). Metalinguistic awareness mediates effects of full biliteracy on third-language reading proficiency in Turkish–German bilinguals. International Journal of Bilingualism, 16, 402–418.

[bibr66-13621688231216869] Reinders H. Benson P. (2017). Research agenda: Language learning beyond the classroom. Language Teaching, 50, 561–578.

[bibr67-13621688231216869] Richards J.C. (2015). The changing face of language learning: Learning beyond the classroom. RELC Journal, 46, 5–22.

[bibr68-13621688231216869] Robinson P. Mackey A. Gass S.M. Schmidt R. (2012). Attention and awareness in second language acquisition. In Gass S.M. Mackey A. (Eds.), The Routledge handbook of second language acquisition (pp. 247–267). Routledge.

[bibr69-13621688231216869] Ross A.S. Rivers D.J. (2018). Emotional experiences beyond the classroom: Interactions with the social world. Studies in Second Language Learning and Teaching, 8, 103–126.

[bibr70-13621688231216869] Schwienhorst K. (2009). The art of improvisation: Learner autonomy, the learner, and (computer-assisted) learning environments. In Kjisik F. Voller P. Aoki N. Nakata Y. (Eds.), Mapping the terrain of learner autonomy: Learning environments, learning communities and identities (pp. 86–117). University of Tampere Press.

[bibr71-13621688231216869] Shin J.Y. Dixon L.Q. Choi Y. (2020). An updated review on use of L1 in foreign language classrooms. Journal of Multilingual and Multicultural Development, 41, 406–419.

[bibr72-13621688231216869] Statistik Austria. (2019). Bildung in Zahlen 2018/19 Schlüsselindikatoren und Analysen. Statistik Austria. Available at: https://www.statistik.at/fileadmin/pages/1865/BiZ_2018_19.pdf

[bibr73-13621688231216869] Statistik Austria. (2023). Bildung in Zahlen 2021/22 Schlüsselindikatoren und Analysen. Statistik Austria. Available at: https://www.statistik.at/fileadmin/user_upload/BiZ-2021-22_Schluesselindikatoren.pdf

[bibr74-13621688231216869] Sulis G. Philp J. (2021). Exploring connections between classroom environment and engagement in the foreign language classroom. In Hiver P. Al-Hoorie A.H. Mercer S. (Eds.), Student engagement in the language classroom (pp. 101–119). Multilingual Matters.

[bibr75-13621688231216869] Svalberg A.M.-L. (2009). Engagement with language: Developing a construct. Language Awareness, 18, 242–258.

[bibr76-13621688231216869] Ticheloven A. Blom E. Leseman P. McMonagle S. (2021). Translanguaging challenges in multilingual classrooms: Scholar, teacher and student perspectives. International Journal of Multilingualism, 18, 491–514.

[bibr77-13621688231216869] Tweed A.D. Reinders H. (2023). Agency and affordances in study abroad. Education Sciences, 13, 327.

[bibr78-13621688231216869] Webb S. Rodgers M.P.H. (2009). Vocabulary demands of television programs. Language Learning, 59, 335–366.

